# Immune checkpoint inhibitor-associated diabetes mellitus: the case series report

**DOI:** 10.3389/fendo.2025.1589630

**Published:** 2025-07-04

**Authors:** Yuhong Ma, Jing Xue, Qianwen Cheng, Hesheng Qian, Yingying Du

**Affiliations:** ^1^ Department of Oncology, The First Affiliated Hospital of Anhui Medical University, Hefei, China; ^2^ Department of Oncology, Fuyang Cancer Hospital, Fuyang, China

**Keywords:** immune checkpoint inhibitor, diabetes mellitus, immune-associated diabetes mellitus, adverse events, clinical manifestations

## Abstract

This report aims to better define the rare adverse event of immune checkpoint inhibitor-associated diabetes mellitus(ICI-DM). We present 10 cases of patients including six of the patients had no prior history of diabetes, while four had varying degrees of pre-existing diabetes. Eight who received anti-PD-1 combination therapy, one who received anti-PD-1 monotherapy, and one who received dual anti-PD-1/CTLA-4 therapy. The mean time from initiation of immunotherapy to the onset of ICI-DM was 245.4 days (median, 149 days; range, 11 to 787 days). Diabetic ketoacidosis (DKA) occurred in 60% (6/10) of the patients, with a median fasting blood glucose level of 25.85 mmol/L (range, 14.76 to 38.23 mmol/L), and all had C-peptide levels below the normal range. Through a retrospective analysis of the clinical data of these 10 patients, we found that monitoring fasting blood glucose and HbA1c is crucial for patients undergoing or having undergone immunotherapy, as rapid pancreatic β-cell destruction can be observed in those who develop ICI-DM, potentially due to disruption of the PD-1/PD-L1 pathway.

## 1 Introduction

We report the case of immune checkpoint inhibitor-associated diabetes mellitus (ICI-DM) and review the literature. The patient has provided their written informed consent for the publication of this manuscript and any identifying images or data. Immune checkpoint inhibitors (ICIs) can activate anti-tumor immune responses by inhibiting the PD-L1 and CTLA-4 immune checkpoint signaling pathways. They have become a mainstay in cancer treatment due to their immunomodulatory effects and survival benefits. However, following the widespread use of ICIs, immune-related adverse events (irAEs) have emerged as a significant concern, affecting multiple organs and systems that can range from mild to life-threatening. IrAEs can lead to discontinuation of treatment, permanent tissue damage or even fatal outcomes. ICI-DM is a relatively rare irAE, with an incidence rate ranging from 0.3% to 3.5% (1). However, there is currently no consensus regarding its diagnosis and management.

During the course of immunotherapy, we conducted best-response assessments for these 10 patients. Among them, six patients demonstrated partial response (PR), two patients exhibited stable disease (SD), and the remaining two patients could not be evaluated for response due to the lack of imaging data.

## 2 Case description

We identified a total of 10 patients who received immunotherapy and were clinically diagnosed with ICI-DM at the First Affiliated Hospital of Anhui Medical University and Fuyang Cancer Hospital from January 2019 to July 2024. Although many studies have discussed the diagnostic criteria for ICI-DM, there is still no unified standard, especially for patients with pre-existing diabetes, accurately identifying ICI-DM remains challenging. Although the latest NCCN guidelines have provided some suggestions, more attention is still needed in actual clinical cases. Therefore, we intentionally divided the patients into two groups: those with diabetes and those without diabetes, for separate discussion.

The diagnostic criteria for ICI-DM are as follows: For patients with a history of diabetes: (i) New-onset fulminant insulin-dependent diabetes or hyperglycemic crisis: Characterized by acute onset and severe metabolic disturbances such as DKA or hyperglycemic hyperosmolar syndrome (HHS). (ii) Deterioration of pre-existing diabetes or prediabetes without other attributable causes: Defined by an increase in fasting blood glucose by more than 50% compared to previous levels, necessitating the use of a second antihyperglycemic agent or insulin, or the occurrence of DKA, new-onset ketonuria, or ketonemia. For patients without diabetes history: For patients in this category, the diagnosis of ICI-DM can be made according to the traditional criteria for diabetes diagnosis (2).

A total of 10 patients were enrolled in this study, and all patients were followed up until December 1, 2024. Among them, four patients (cases 3, 4, 8, 9) unfortunately passed away due to disease progression. The remaining six patients had a mean overall survival of 691.3 days from the initiation of immunotherapy to the last follow-up (median, 654.5 days; range, 127 to 1326 days).

### 2.1 Non-diabetic group

Six patients without a history of diabetes were enrolled. Only one patient received monotherapy with an immune checkpoint inhibitor, while the remaining five patients were treated with a combination of immunotherapy and chemotherapy or targeted therapy, and all patients underwent anti-PD-1 immunotherapy. Case 1 presented to the emergency department due to anorexia, fatigue, and severe nausea and vomiting, with a fasting blood glucose level of 22.77 mmol/L upon admission. Case 2 was admitted due to sudden loss of consciousness accompanied by nausea and vomiting, with a fasting blood glucose level of 20.16 mmol/L. Case 6 was found to have urinary ketones (2+) and a fasting blood glucose level above the normal range on routine urinalysis during the course of immunotherapy. The remaining three patients developed varying degrees of polydipsia, polyuria, and polyphagia during immunotherapy, leading to their identification. No other specific positive physical signs were observed. The patients denied any family history of hereditary diseases and any history of corticosteroid use. In this cohort, two patients (case 1, 2) tested negative for a panel of diabetes-related autoantibodies, which included islet cell antibodies (ICA), anti-glutamic acid decarboxylase antibodies (anti-GAD or GADA), insulin autoantibodies (IAA), tyrosine phosphatase-like protein antibodies (IA-2A), and zinc transporter 8 antibodies (ZnT8Ab). Following treatment with continuous intravenous insulin infusion or insulin pump therapy, the blood glucose levels of patients in this group were able to return to the normal range. However, upon cessation of antihyperglycemic treatment (oral medications or subcutaneous insulin injections), blood glucose levels could rise again, necessitating long-term dependence on exogenous insulin to maintain stable blood glucose levels. During the follow-up period, despite regular subcutaneous insulin injections, fasting blood glucose (BS) levels continued to fluctuate. In this group, two patients(case 1,2) had hypothyroidism. The average time to onset of ICI-DM from the initiation of ICIs was 349 days (median 229.5 days; range 50–787 days) (**Table 1**).

**Table 1:** Patient information and laboratory tests at initial diagnosis in the non-diabetes group.

Patient/Characteristic	Case 1	Case 2	Case 3	Case 4	Case 5	Case 6
Demographics						
Gender	Female	Female	Male	Male	Male	Male
Age	54	53	55	45	61	70
Primary tumor	Renal cancer	Lung cancer	Gastric cancer	Lung cancer	Renal cancer	Lung cancer
Treatment	Tislelizumab and Axitinib	Sintilimab	Karelizumab, nab-Paclitaxel and Tegeo	Nivolumab, Pemetrexed and Carboplatin	Tislelizumab and Axitinib	Karelizumab, Paclitaxel liposome and Carboplatin→Karelizumab
After use ICIs						
Tumor response	PR	/	PR	PR	SD	/
Time to diagnosis of ICI-DM/(days)	289	670	128	787	50	170
Treatment of ICI-DM	Aspart insulin(three times daily) + Glargine insulin(once nightly)	Aspart insulin(three times daily) + Glargine insulin(once nightly)	Gliclazide+Metformin(three times daily)	Aspart insulin(three times daily) + Glargine insulin(once nightly)	Aspart 30 insulin(once daily)	Aspart insulin(three times daily) + Glargine insulin(once nightly)
Re-challenge	No	No	No	No	Yes	No
Other irAEs	hypothyroidism	hypothyroidism	No	No	No	No
OS/(days)	≥800	≥1326	563	1690	≥509	≥263
Laboratory data						
Fasting glycemia^①^/(mmol/L)	6.55	6.35	9.15	6.27	5.50	4.65
Fasting glycemia^②^/(mmol/L)	22.77	20.16	18.00	18.70	27.00	14.76
HbA1c/(%)	9.00	8.40	/	6.60	10.10	9.70
0hC-peptide/(ng/ml)	0.06	0.09	0.76	<0.01	0.13	0.19
Urine ketone	2+	3+	–	–	–	2+

The tumor response is assessed based on the Response Evaluation Criteria in Solid Tumors (RECIST 1.1), PR, partial response; SD, stable disease; OS, overall survival. Fasting glycemia ^①^:the most recent documented fasting blood glucose result before ICI-DM. Fasting glycemia ^②^:the fasting blood glucose in patients presenting with ICI-DM. Reference for laboratory data: Fasting glycemia: 3.89-6.11mmol/L, HbA1c/(%): 4-6%, C-peptide(0h): 0.81-3.85ng/ml, Urine ketone: -./ indicates that the result was not followed up. “→” indicates that the patient received immunotherapy monotherapy maintenance after the combination of immunotherapy and chemotherapy.

### 2.2 Diabetic group

We included four patients with a history of T2DM. Among them, Case 8 and Case 10 achieved glycemic control through long-term subcutaneous insulin injections, while Case 7 and Case 9 managed their blood glucose levels with regular oral hypoglycemic agents. All four of these patients had previously well-controlled blood glucose levels. Four patients received immunotherapy combined with chemotherapy or targeted therapy; three patients received anti-PD-1 immunotherapy, while one patient were treated with a combination of anti-PD-1/CTLA-4 immunotherapy. Case 7 presented with dry mouth, polyuria, fatigue, accompanied by dizziness, palpitations, and chest tightness, the fasting blood glucose level was measured at 38.23 mmol/L. Despite aggressive treatment with oral hypoglycemic agents in combination with subcutaneous insulin injections, the patient’s blood glucose levels remained poorly controlled. Case 8 primarily manifested with nausea and vomiting, accompanied by gradually worsening dizziness and fatigue. Case 9 suddenly developed deep and labored breathing, along with palpitations and somnolence during hospitalization, with a blood glucose level of 30.54 mmol/L. Case 10 was identified due to anorexia accompanied by discomfort in the upper abdomen. No other specific positive physical signs were observed. The patients denied any family history of hereditary diseases and any history of corticosteroid use. In this cohort, only two patients (case 7, 10) had previously undergone diabetes autoantibody testing, and the results were negative for both. In this cohort of patients, the existing antihyperglycemic regimens were ineffective at maintaining blood glucose levels within the normal range following the development of ICI-DM, necessitating a change in their diabetes management plans (either by increasing the dosage of subcutaneous insulin injections, or by adding other oral hypoglycemic agents). Even after achieving stable blood glucose control, continuation of the original antihyperglycemic regimen was not feasible. In this group, only one patients(case 9) had hypothyroidism. The mean time to onset of ICI-DM from the initiation of ICIs was 90 days (median 67 days range 11–215 days) (**Table 2**).

**Table 2:** Patient information and laboratory tests at initial diagnosis in the diabetes group.

Patient/Characteristic	Case7	Case8	Case9	Case10
Demographics				
Gender	Female	Male	Male	Female
Age	76	68	70	66
Past history	ten-year diabetes	ten-year diabetes	fifteen-year diabetes	ten-year diabetes
Primary tumor	Gastric cancer	Lung cancer	Pancreatic	Bladder cancer
Treatment	Nivolumab, nab-Paclitaxel and Carboplatin→Nivolumab	Karelizumaband nab-Paclitaxe → karelizumab	PD-1/CTLA-T, nab-Paclitaxe and Gemcitabine	Nivolumab, Gemcitabine and Carboplatin
After use ICIs				
Tumor response	PR	PR	PR	SD
Time to diagnosis of ICI-DM/(days)	215	96	11	38
Treatment of ICI-DM	Aspart insulin(three times daily) + Glargine insulin(once nightly)	Aspart insulin(three times daily) + Glargine insulin(once nightly)	Aspart insulin(three times daily) + Glargine insulin(once nightly)	Aspart insulin(three times daily) + Glargine insulin(once nightly)
Re-challenge	Yes	Yes	Yes	Yes
Other irAEs	No	No	hypothyroidism	No
OS/(days)	≥1123	781	956	≥127
Laboratory data				
Fasting glycemia^①^/(mmol/L)	8.68	12.51	9.82	9.06
Fasting glycemia^②^/(mmol/L)	38.23	28.93	30.54	16.60
HbA1c/(%)	11.10	9.60	/	8.00
0hC-peptide/(ng/ml)	<0.01	0.18	0.38	0.13
Urine ketone	2+	2+	3+	–

“→” indicates that the patient received immunotherapy monotherapy maintenance after the combination of immunotherapy and chemotherapy.

## 3 Discussions

The specific mechanisms underlying ICI-DM are still being elucidated. There is already evidence indicating that the occurrence of ICI-DM may be associated with genetic susceptibility (3), among which the human leucocyte antigen class II (HLA-II) genes are the primary genetic susceptibility factors for the development of ICI-DM (4). Some studies have found that the HLA susceptibility genotypes for ICI-DM overlap with those for type 1 diabetes mellitus (T1DM) and fulminant diabetes (FD), but these susceptibility genotypes cannot account for all occurrences of ICI-DM (5–7). Liu Yichen et al. (8) included HLA genotypes from eight Chinese patients with ICI-DM, and found that 75.0% of patients carried HLA susceptibility genotypes, whereas 4.0% harbored protective genotypes. This indicates that the genetic factors underlying ICI-DM differ from classical diabetes. Additionally, abnormal expression of PD-L1 in pancreatic β-cells may contribute to the development of ICI-DM. Studies have shown that the majority of ICI-DM cases develop following treatment with PD-1 or PD-L1 inhibitors, rather than CTLA-4 inhibitors, indicating a potential link to the aberrant expression of PD-L1 by pancreatic β-cells. Concurrently, research has indicated that PD-L1 expression is increased in the pancreatic β-cells of aged mice and mice with islet immune infiltration, while the disruption of the PD-1/PD-L1 signaling pathway can induce diabetes in the non-obese diabetic model mice (9). In humans, PD-L1 is selectively expressed on the surface of functional pancreatic β-cells among patients with type 1 diabetes mellitus (T1DM), but not those in healthy individuals (10). One case report of pancreatic biopsies from ICI-DM patients revealed extensive CD8+ T-lymphocyte infiltration and a paucity of functional β-cells, with the remaining β-cells lacking PD-L1 expression (11). This indicates that ICIs may disrupt the PD-1/PD-L1 pathway in pancreatic β-cells, thereby contributing to islet dysfunction. However, further exploration and elucidation of underlying mechanisms are required.

ICI-DM is a rare yet severe irAE. Currently, there are no unified diagnostic criteria for ICI-DM, and the practical difficulties in obtaining pancreatic tissue preclude definitive histological diagnosis. Therefore, in this retrospective study of ICI-DM patients, we attempt to provide empirical insights into the diagnosis of ICI-DM, especially for patients with a pre-existing history of diabetes. For patients with a history of diabetes, the diagnosis of ICI-DM presents a challenge. Although the latest NCCN guidelines propose diagnostic criteria for ICI-DM, suggesting that a fasting blood glucose level greater than 11.1 mmol/L or a random blood glucose level greater than 13.9 mmol/L, or a history of type 2 diabetes with a fasting/random blood glucose level greater than 13.9 mmol/L, should prompt consideration of measuring autoantibodies and C-peptide levels to assess and classify ICI-DM. Compared with the latest NCCN guidelines, the diagnostic criteria proposed in this study first stratify patients based on whether they have a history of diabetes and then apply different diagnostic standards to each group. This stratified approach enables more precise identification of ICI-DM patients, especially those with a pre-existing history of diabetes, facilitating differentiation between ICI-DM and changes in pre-existing diabetes. Secondly, the diagnostic criteria proposed in this study emphasize comparing current blood glucose levels with those when the patient’s condition was stable. If the blood glucose level has increased by more than 50% from the previous level, ICI-DM should be considered. This method takes individual differences into account, avoiding potential misdiagnoses that may arise from fixed numerical standards. Moreover, this study not only focuses on changes in blood glucose levels but also integrates clinical manifestations (such as acute onset, diabetic ketoacidosis, etc.), providing a more comprehensive basis for diagnosis.

ICI-DM is characterized by its acute onset, with approximately 71% of patients initially presenting with DKA, accompanied by low levels of glycated hemoglobin (12). In the non-diabetes group, three patients were considered to have potentially developed DKA. These patients were mostly admitted due to sudden severe nausea and vomiting, along with altered mental status or positive urine ketone test results. After timely fluid resuscitation, continuous intravenous insulin infusion, correction of electrolyte imbalances, and dynamic monitoring, the patients’ symptoms gradually improved. Subsequently, they were transitioned to a subcutaneous basal insulin regimen to maintain stable blood glucose levels. In the diabetes group, patients were usually identified due to a sudden exacerbation of symptoms such as dizziness, somnolence, and nausea and vomiting. The existing blood glucose control regimens for these three patients were unable to rapidly normalize their blood glucose levels. After intensive monitoring, fluid resuscitation, and insulin therapy, and adjustments to their original blood glucose management plans based on their current blood glucose levels, the patients’ blood glucose levels were eventually maintained within the normal range. Given the acute presentation and severity of DKA, it is essential to regularly monitor the fasting blood glucose levels of patients undergoing immunotherapy to prevent its occurrence. For patients who have already developed DKA, timely medical intervention is necessary, and the treatment regimen can follow the management guidelines for DKA caused by T1DM.

The management strategies for ICI-DM mainly consist of acute phase management and long-term management. For acute phase management, fluid resuscitation with isotonic saline is crucial, administered based on dehydration severity, starting rapidly then slowing. Continuous intravenous insulin infusion, typically at 0.1 U/kg per hour (4–6 U/h for adults, max 10 U/h), is the main method for lowering blood glucose. Potassium supplementation and blood gas monitoring are essential, with efforts to identify and eliminate DKA triggers. Most ICI-DM patients require lifelong insulin therapy post-acute phase, the treatment goal is to prevent DKA and avoid severe hypoglycemia, which can be achieved through relatively low doses of basal insulin (0.05–0.1 U/kg/day). In addition, through individualized insulin regimens and dynamic management, combined with patient education and support, patient prognosis can be effectively improved. Long-term management focuses on regular monitoring and complication screening. Patients should test fasting and postprandial blood glucose 2–3 times weekly initially, reducing frequency once stable, with at least one HbA1c test per month. Continuous glucose monitoring may be considered for those with significant fluctuations or poor control. Regular C-peptide level testing (every 3–6 months) helps assess pancreatic β-cell function and adjust treatment. Complication screening includes annual fundoscopic exams, biannual renal function tests, and annual neuropathy assessments.

The occurrence of ICI-DM may be associated with better treatment outcomes. Several reports have suggested that patients who develop ICI-DM often exhibit significant antitumor treatment efficacy, with a disease control rate as high as 76.9% (13). Emma S. Scott et al. (14), in their study on immune checkpoint inhibitors and other endocrine adverse events, found that among the patients who developed endocrine adverse events, 16 (52%) had a response to ICIs, with 11 (32%) showing stable disease (SD) and 5 (16%) showing progressive disease (PD). In our study, among the 10 enrolled patients, aside from two who could not be evaluated for antitumor efficacy due to a lack of imaging data, 6 showing partial response (PR) and 2 showing stable disease (SD). Although our study is limited by its small sample size, it cannot be denied that there is a certain correlation between the occurrence of ICI-DM and the best antitumor response in patients. These studies collectively suggest that the occurrence of ICI-DM may be associated with favorable antitumor outcomes, and it cannot be ruled out that this association may be related to long-term treatment with ICIs for tumors. Further clinical research is needed to elucidate this relationship.

The issue of whether to discontinue immunotherapy after the occurrence of hyperglycemia is a highly debated topic in clinical practice. Additionally, there is currently no clear consensus on whether it is safe to restart ICI treatment after blood glucose levels have been controlled. Studies have reported cases of autoimmune diabetes occurring after immunotherapy, where the autoimmune diabetes was not successfully reversed despite immunomodulatory treatment with high-dose corticosteroids (the standard treatment for irAEs) (15). Another study (16) found that among patients with ICI-DM who had their blood glucose controlled, 36.9% (69/187) were re-challenged with ICIs, and at the last follow-up, 89.9% (62/69) of these patients were still receiving immunotherapy. In our study, among the 10 patients enrolled, 50% (5/10) of the patients chose to undergo rechallenge with ICIs and continued to receive immunotherapy at the last follow-up. Based on the above findings, for patients who develop ICI-DM, restarting immunotherapy after stable blood glucose control may be more beneficial for prognosis. However, further clinical studies are needed to confirm.

## 4 Limitations and future directions

This study, which preliminarily explored the clinical features and mechanisms of immune checkpoint inhibitor-related diabetes mellitus (ICI-DM), has several limitations. The small sample size of 10 patients restricts the statistical power and generalizability of the results. The retrospective design may introduce biases in data completeness and accuracy, and the mechanistic analysis lacks direct experimental validation. The short follow-up period limits insights into long-term outcomes and complications.

Future studies must expand the sample size and conduct multicenter, prospective research to enhance reliability and generalizability. Standardizing clinical data collection and management to ensure completeness and accuracy is crucial. Additionally, integrating experimental research methods to explore the pathogenesis of ICI-DM will strengthen the basis for clinical diagnosis and treatment.

## Data Availability

The original contributions presented in the study are included in the article/Supplementary Material. Further inquiries can be directed to the corresponding authors.
